# *AdNAC20* Regulates Lignin and Coumarin Biosynthesis in the Roots of *Angelica dahurica* var. *Formosana*

**DOI:** 10.3390/ijms25147998

**Published:** 2024-07-22

**Authors:** Wenjie Qu, Wenjuan Huang, Chen Chen, Jinsong Chen, Lin Zhao, Yijie Jiang, Xuan Du, Renlang Liu, Yinyin Chen, Kai Hou, Dongbei Xu, Wei Wu

**Affiliations:** College of Agronomy, Sichuan Agricultural University, Chengdu 611130, China; qwj1941428057@163.com (W.Q.); 15754368373@139.com (W.H.); doublechen_swun@yeah.net (C.C.); chjs1993@163.com (J.C.); linzhao0518@outlook.com (L.Z.); jyj177ky@163.com (Y.J.); 15729872162@163.com (X.D.); liurenlang2023@163.com (R.L.); 14227@sicau.edu.cn (Y.C.); hking@sicau.edu.cn (K.H.); xudongbei2006@126.com (D.X.)

**Keywords:** NAC transcription factor, early bolting, CRISPR/Cas9 technology, transcriptome data analysis

## Abstract

*Angelica dahurica* var. *formosana* (*ADF*), which belongs to the Umbelliferae family, is one of the original plants of herbal raw material Angelicae Dahuricae Radix. *ADF* roots represent an enormous biomass resource convertible for disease treatment and bioproducts. But, early bolting of *ADF* resulted in lignification and a decrease in the coumarin content in the root, and roots lignification restricts its coumarin for commercial utility. Although there have been attempts to regulate the synthesis ratio of lignin and coumarin through biotechnology to increase the coumarin content in ADF and further enhance its commercial value, optimizing the biosynthesis of lignin and coumarin remains challenging. Based on gene expression analysis and phylogenetic tree profiling, *AdNAC20* as the target for genetic engineering of lignin and coumarin biosynthesis in *ADF* was selected in this study. Early-bolting *ADF* had significantly greater degrees of root lignification and lower coumarin contents than that of the normal plants. In this study, overexpression of *AdNAC20* gene plants were created using transgenic technology, while independent homozygous transgenic lines with precise site mutation of *AdNAC20* were created using CRISPR/Cas9 technology. The overexpressing transgenic ADF plants showed a 9.28% decrease in total coumarin content and a significant 12.28% increase in lignin content, while knockout mutant plants showed a 16.3% increase in total coumarin content and a 33.48% decrease in lignin content. Furthermore, 29,671 differentially expressed genes (DEGs) were obtained by comparative transcriptomics of OE-*NAC20*, KO-*NAC20*, and WT of *ADF*. A schematic diagram of the gene network interacting with *AdNAC20* during the early-bolting process of *ADF* was constructed by DEG analysis. *AdNAC20* was predicted to directly regulate the transcription of several genes with SNBE-like motifs in their promoter, such as MYB46, C3H, and CCoAOMT. In this study, *AdNAC20* was shown to play a dual pathway function that positively enhanced lignin formation but negatively controlled coumarin formation. And the heterologous expression of the *AdNAC20* gene at *Arabidopsis thaliana* proved that the *AdNAC20* gene also plays an important role in the process of bolting and flowering.

## 1. Introduction

Angelicae Dahuricae Radix (Baizhi in China) is the dried root of *Angelica dahurica* (Fisch. Ex Hoffm.) Benth. et Hook. f. or *Angelica dahurica* (Fisch. ex Hoffm.) Benth. et Hook. f. var. *formosana* (Boiss.) Shan et Yuan [1]. The latter is the original plant of the Chinese medicine Baizi in Sichuan Province, China. It is believed to treat acne, ulcers, rheumatism, *Angelica dahurica* var. *formosana* (*ADF*) lupus erythematosus, headache, toothache, etc. [2]. However, early bolting of (*ADF*) occurs during the cultivation process, during which plants prematurely transition from vegetative growth to the reproductive growth stage, resulting in the hollowing and lignification of the roots, ultimately leading to a decrease in the weight and medicinal and nutritional value of the roots [3,4]. In addition to changes in roots, early-bolting plants are accompanied by the development of flower organs and an increase in plant height [5]. Similar findings have also been reported for plants such as *Peucedanum praeruptorum* Dunn (*P. praeruptorum*), *Angelica sinensis*, and *Saposhnikovia divaricata* of the Umbelliferae family, which also increase lignin and lignan contents in the xylem and decrease biologically active ingredients, preventing the collection of roots for medicinal purposes [6]. Preventing the early bolting of *A. angelica* is very important for improving its quality and yield.

The effective components of *ADF* are mainly coumarins, especially furocoumarins, such as imperatorin, isoimperatorin, hydrated and oxidated imperatorin, byakangclicol, angelicin, etc. [4,7]. Coumarins are located mainly in the oil tubes of the root phloem [3]. Phenylpropanoids, including lignin, lignans, and coumarins, contribute to all aspects of plant responses to biotic and abiotic stimuli [8,9]. Phenylalanine is catalyzed by phenylalanine ammonia lyase (PAL), cinnamate-4 hydroxylase (C4H), and 4-coumaryl-CoA ligase (4CL) to produce cinnamic acid, p-hydroxycoumaric acid (pCA), and coumaryl-CoA in most plants. The initial three steps of the phenylpropanoid biosynthesis pathway are mandatory and provide the basis for the resulting lignin, coumarin, and other phenylpropanoids [8,9]. *CCoAOMT* not only participates in lignin biosynthesis but also leads to a reduction in the coumarin scopoletin in *Arabidopsis thaliana* (*A. thaliana*) roots [10,11]. According to comparative transcriptomics coupled with coexpression-associated analysis of *P. praeruptorum*, coumarin biosynthetic genes include *PAL*, *C4H1*, *C3H*, *HCT*, *COMT*, *CCoAOMT*, *AS*, *PS1*, *BMTs*, *PRXs*, and three peroxidases involved in lignin biosynthesis [6,12]. These genes are downregulated during bolting and ultimately reduce the formation of coumarins [6,12]. The overexpression of *AaCAD* in *Artemisia annua* enhances lignin and coumarin formation but decreases the levels of artemisinin, artemisinin B, and other sesquiterpenes [13].

The expression of coumarin-related genes and lignin-related genes also changes significantly during the early bolting of *ADF* [4]. According to the statistical analysis of the differential expression of transcription factors between early-bolting and normal *ADF* plants, the early bolting of *ADF* involves many gene families, including the MADS-box family, NAC family, MYB family, and WRKY family, among which the NAC family is one of the plant-specific transcription factor (TF) families and one of the largest families of transcription factors.

The NAC family plays an important role in plant growth and development. The NAC family is involved in the formation of xylem [14]. At present, one of the best-studied events of xylem formation is secondary wall thickening [15]. Secondary xylem usually has a thickened secondary cell wall formed by a variety of polymers, such as lignin, cellulose, and hemicellulose [14]. The N-terminal NAC domain of NAC family genes is composed of 150–160 aa, which are highly conserved and divided into conserved subdomains A, B, C, D, and E, among which D and E are DNA binding regions, and subdomain A plays a role in the formation of protein dimers [16,17]. The C-terminus is a variable transcriptional regulatory domain that regulates gene expression by binding to proteins or DNA [17]. *SND*, *NSTs*, *VNDs*, *ANAC005*, *VNI2*, and *XND1* of *A. thaliana* participate in xylem development, secondary wall and cell expansion, among which *VND7* has been identified as a master switch of xylem vessel differentiation [18], and *XND1* and *VNI2* have been identified as inhibiting xylem vessel formation [19,20]. *VNI2* and *VNDs* interact to regulate xylem vessel formation [19]. *VND1–7* was identified as the master switches controlling the terminal events of xylem vessel formation. SLR1 of the NAC family interacts with the GA signaling inhibitor DELLA protein to regulate secondary wall cellulose biosynthesis in rice [21]. NAC proteins are also involved in the transcriptional regulation of plant flowering and stress [14,22,23].

Through CDD 3.17, Pfam 32.0, and MEGA 6.0 screening, verification, and identification, 75 NAC transcription factor sequences were obtained from *ADF*, and genes related to early bolting were screened. Here, we described a series of experimental findings suggesting that *AdNAC020* is involved in the biosynthesis of coumarin and lignin during the early bolting of *ADF*.

## 2. Results

### 2.1. Physiological Changes during Early Bolting of ADF

**Figure 1:**
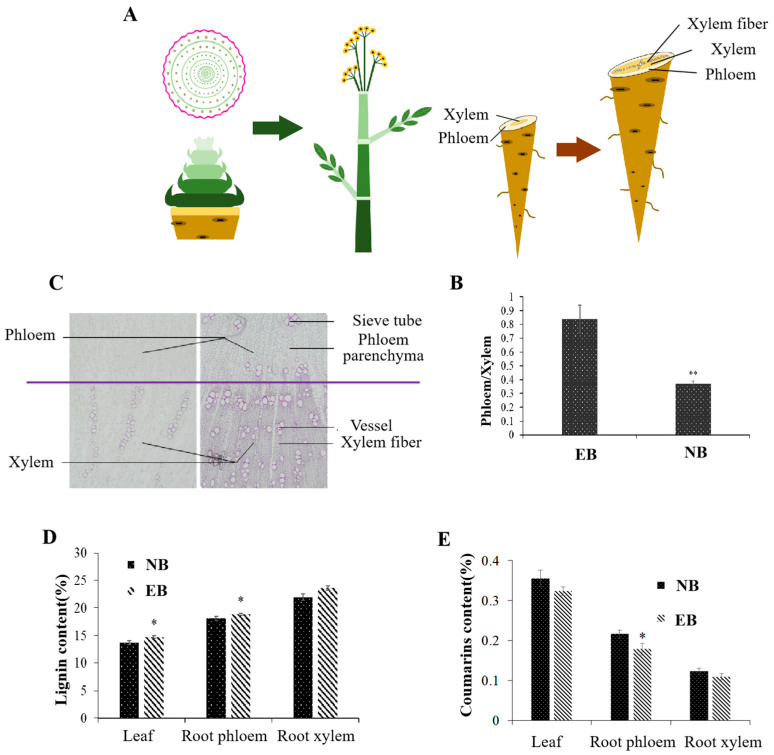
Physiological changes during early bolting of *ADF*. (**A**) Development of flowering and bolting. (**B**) Root phloem/xylem ratio of early-bolting and normal plants. (**C**) The right panel shows early-bolting plants, and the left panel shows normal plants. (**D**) Lignin content of early-bolting and normal plants. (**E**) Coumarin content of early-bolting and normal plants. EB, early-bolting *ADF*; NB, non-bolting *ADF*. * Indicates extremely significant difference (*p* < 0.05); ** indicates extremely significant difference (*p* < 0.01).

Through the observation of the morphological characteristics of *ADF*, the transition from vegetative growth to the reproductive growth stage of early-bolting plants occurred when the plants reached nine or ten compound leaves. This indicated flower physiological initiation, axis elongation in the early stages of early bolting, stem internode elongation in the late stages of early bolting, and increased plant height. Early bolting of *ADF* significantly reduced the phloem/xylem ratio of roots (Figure 1A,B); increased the number of sieve tubes, vessels and fibers; increased the thickness of secondary cell walls; and eventually promoted the hollowing and lignification of roots (Figure 1C). The contents of lignin in early-bolting plants, were significantly higher than those of normal plants, especially in the root phloem. (Figure 1D). However, the contents of coumarins, which are the biologically active compounds of early-bolting plants, were significantly lower than those of normal plants, especially in the root phloem (Figure 1E). In conclusion, the early bolting of *ADF* affected internode elongation and flower initiation, accelerated root lignification, and the proportion of the phloem of highly enriched biologically active compounds was reduced. Ultimately, the yield and quality of *ADF* were seriously reduced.

### 2.2. Cloning, Sequence Analysis and Expression Profiles of AdNAC20 in ADF

#### 2.2.1. Cloning and Sequence Analysis of *AdNAC20*

According to a previous report on the difference between early-bolting (EB) and non-bolting (NB) roots of *ADF* based on transcriptome sequencing, many DEGs of the NAC family participate in the process of early bolting of *ADF*. Seventy-five NAC proteins were predicted to be involved in *ADF* [24]. Then, a phylogenetic tree was constructed for 75 AdNACs of *ADF* and 138 ANACs of *A. thaliana*, in which the differential gene *AdNAC020* was located in the *SENU5* subfamily, in which *VNI2* inhibits xylem vascular differentiation [19].

**Figure 2:**
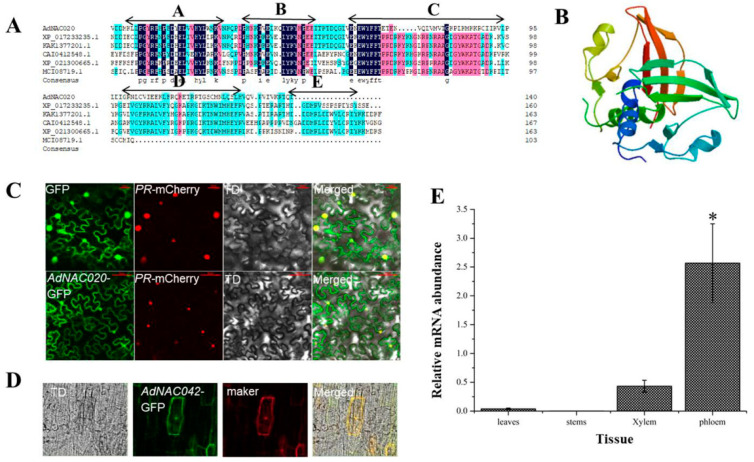
Sequence analysis of *AdNAC20. (***A**) Homology analysis of the protein encoded by the *AdNAC020* gene. (**B**) Protein structure predicted by SWISS-MODEL homologous modeling. (**C**) Subcellular localization analysis of *AdNAC020* in *N. benthamiana*. (**D**) Subcellular localization analysis of *AdNAC020* in *ADF* leaves. (**E**) Tissue expression analysis of *AdNAC020*. GFP, blank control; PR mCherry, nuclear localization marker; TD, bright; merged, combination map; marker, nuclear and cytomembrane localization marker. * Indicates extremely significant difference (*p* < 0.05).

In this investigation, the *AdNAC20* cDNA sequence was cloned from *ADF* and was found to be 528 bp in length, encoding a 174 aa protein (Table S4). A motif analysis with MEME version 5.0.5 software revealed that the *AdNAC20* protein had a conserved NAC domain (https://meme-suite.org/meme/tools/meme (accessed on 15 April 2019)), but the NAC domain of *AdNAC20* included five subdomains, A to E (Motif2, Motif5, Motif1, Motif3 and Motif4), The high stability of the A, B, and C subdomains, especially the A subdomain, may maintain the structural stability and some functional aspects of the NAC family stability. The D and E subdomains are not conserved, suggesting that *AdNAC20* may play more diverse functions (Figure 2A). The physicochemical properties and secondary helical structure of the *AdNAC20* protein were analyzed by bioinformatics (Tables S5 and S6), and its tertiary structure was predicted by SWISS-MODEL homologous modeling (Figure 2B). Sequence comparative analysis revealed that *AdNAC20* was closely related to *Daucus carota* L. var. *sativa* Hoffm. (XP_017233235.1, 77.61%), *Heracleum hemsleyanum* Diels. (KAK1377201.1, 77.61%), *Linum usitatissimum* L. (CAI0412548.1, 62.30%), *Malva sinensis* Cavan. (XP_021300665.1, 59.02%), and *Galium odoratum* (L.) Scop. (MCT08719.1, 53.73%) (Figure 2A).

As predicted by subcellular localization by CELLO v.2.5 (http://cello.life.nctu.edu.tw/cello.html (accessed on 1 May 2019)), the subcellular localization experiments of *N. benthamiana* and *ADF* plants showed that AdNAC020 was located in the nucleus and other parts of the cells (Figure 2C,D). According to qRT-PCR analysis, the expression of *AdNAC20* was higher in the root phloem than in the leaf, stem, and root xylem of the *ADF* treatment group *(*Figure 2E). These results suggested that *AdNAC20* might be involved in the development and function of *ADF* roots.

#### 2.2.2. Transient *AdNAC20* Expression in ADF Roots

**Figure 3:**
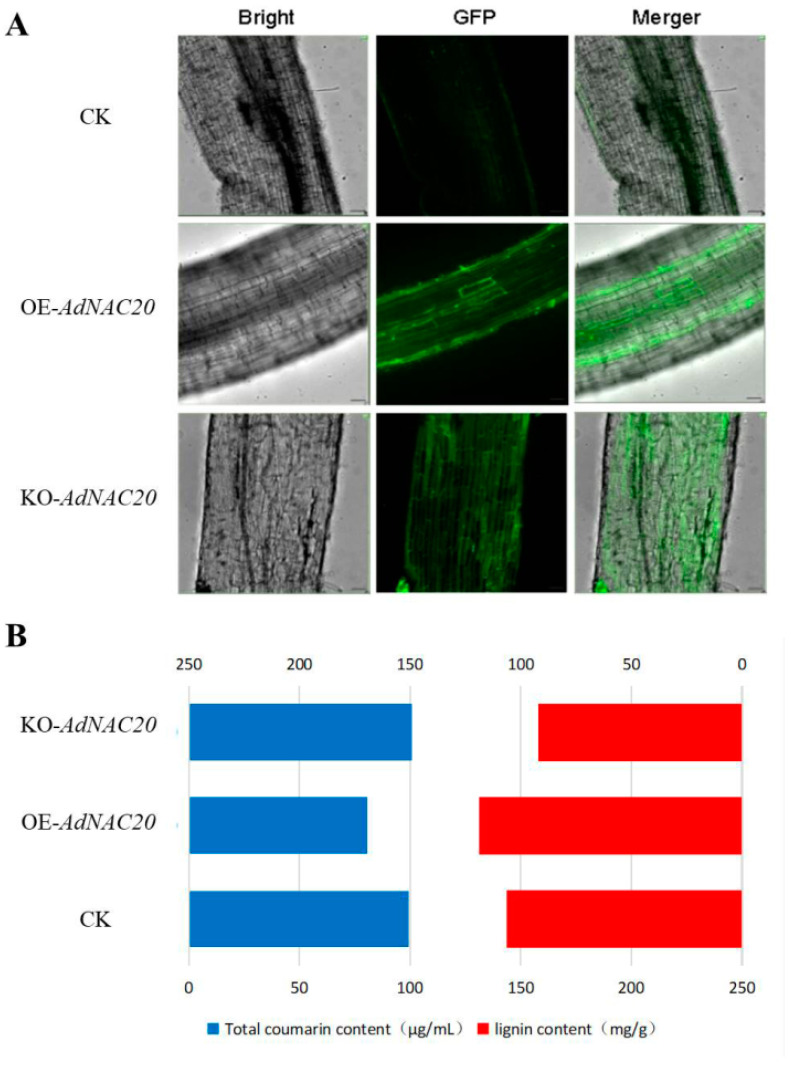
Instantaneous transformation of the *ADF* root system by *Agrobacterium*-mediated root leaching. (**A**) Instantaneous conversion fluorescence map after root immersion. (**B**) Lignin and total coumarin contents in roots after instantaneous transformation of each bacterial solution. OE-*NAC20*, *AdNAC20*-overexpressing plants of *ADF*; KO-*NAC20, AdNAC20* mutant plants of *ADF*; CK, wild-type plants of *ADF*.

The *AdNAC20* gene was transiently expressed in the roots of *ADF* using 35S::*AdNAC20* and *AdNAC20-Cpf1 Agrobacterium* infiltration methods to verify its biological function (Figure 3A). The infiltrated roots displayed strong fluorescence, indicating that the *AdNAC20* gene was successfully expressed. The cross-sections of the infiltrated roots were subjected to specific phloroglucinol staining, which revealed that the degree of root lignification decreased in the following order: OE-*NAC20* > CK > KO-*NAC20 (*Figure 3). Compared with that of the non-infiltrated control, the lignin content of OE-*NAC20* significantly increased by 11.80%, and the coumarin content significantly decreased by 18.65%. In contrast, compared with that of the control, the lignin content of KO-*NAC20* significantly decreased by 13.28%, and the coumarin content increased by 1.49% (Figure 3B). Transient expression of *AdNAC20*-GFP revealed that *AdNAC20* was involved in lignin and coumarin biosynthesis in *ADF* roots.

### 2.3. Heterogeneous Expression of AdNAC20 in A. thaliana

#### 2.3.1. *AdNAC20* Regulates Plant Height and Other Phenotypes in the Aerial Parts of *A. thaliana*

Overexpression of the *AdNAC20* gene in *A. thaliana* complemented the plant height phenotype, thick stems, number of first-order branches and other phenotypes of *At nac20*. 15 35S: *At NAC200* plants, seventeen *At nac20* plants and three RC *At NAC20* plants from T3 homozygous lines of *A. thaliana* were identified via qRT-PCR. Compared with those in wild-type (WT) plants, the development of bolting and flowering in *AdNAC20*-overexpressing lines of *A. thaliana* were flowering 6 days earlier, while knockout of the *AdNAC20* gene resulted in a delay of 4–6 days, the reverse-complemented lines were flowering 5 days earlier. In addition, other phenotypic traits of RC *At NAC20* were consistent with those of 35S:*At NAC20*. For instance, the leaves of 35S:*At NAC20* and RC *At NAC20* plants were significantly shorter than those of WT plants, but the leaves of *At nac20* plants were taller than those of WT plants. RC *At NAC20* complemented the height of *At nac20* plants. These results showed that the *AdNAC20* gene promoted the transformation from vegetative growth to reproductive growth, shortened the vegetative growth period, and reduced the accumulation of nutrients. In summary, the heterologous expression of the *A. thaliana* gene proved that the *AdNAC20* gene plays an important role in the process of bolting and flowering.

#### 2.3.2. *AdNAC20* Regulates Root Phenotype and Lignin Content in *A. thaliana*

**Figure 4:**
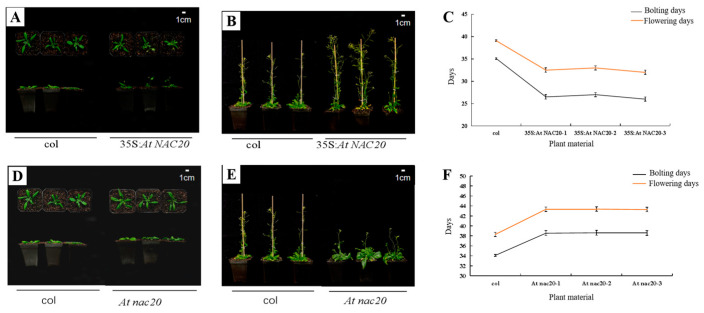

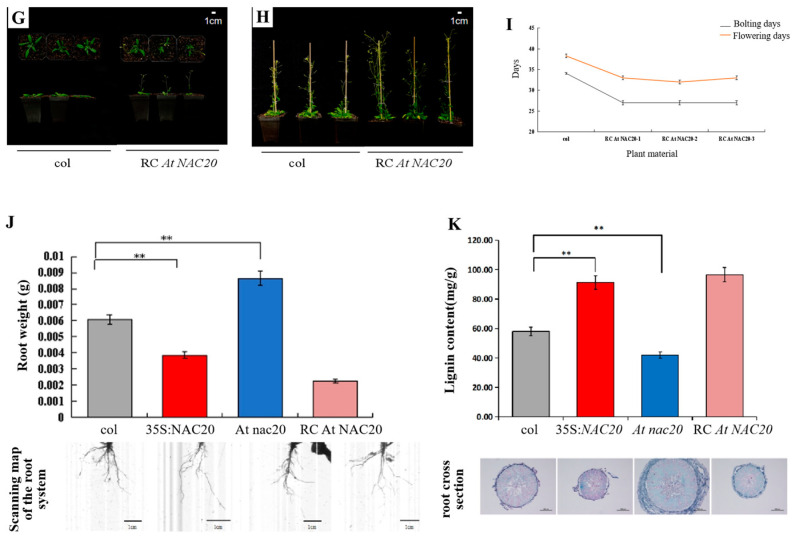
Expression of *AdNAC20* in *A. thaliana* and its phenotype. (**A**) *A. thaliana* plants overexpressing *AdNAC20* at the early-flowering stage on day 33. (**B**) Phenotypes of *A. thaliana* plants overexpressing *AdNAC20* at the late-flowering stage of 44 days. (**C**) Statistics of flowering days for bolting *A. thaliana* plants overexpressing *AdNAC20*. (**D**) The early-flowering phenotype of *A. thaliana* plants with *AdNAC20* deletion on day 33. (**E**) Phenotypes of *A. thaliana* plants with *AdNAC20* deletion at the late-flowering stage of 44 days. (**F**) Statistics of flowering days for bolting *A. thaliana* plants with the *AdNAC20* gene deletion mutant. (**G**) The *AdNAC20* deletion mutant supplemented the early-flowering phenotype of *A. thaliana* at 33 days. (**H**) The phenotype of *A. thaliana* plants at the late-flowering stage 44 days after *AdNAC20* deletion. (**I**) Statistics on flowering days of bolting *A. thaliana* plants supplemented with the *AdNAC20* gene deletion mutant. (**J**) Root weight statistics and root scanning of *A. thaliana* seedlings after 60 days. (** indicates highly significant differences.) (**K**) Observation of lignin content in *A. thaliana* after 60 days of seeding and root cross-cut saffron–solid green staining. Red indicates lignified cell tissue. Green indicates cellulose cell tissue. 35S:*NAC20*, *AdNAC20* overexpression lines of *A. thaliana* plants; RC *At NAC20*, *AdNAC20* reverse-complemented lines of *A. thaliana* plants; *At nac20*, *AdNAC20* mutant lines of *A. thaliana* plants; col, wild-type of *A. thaliana* plants (Col-0). (** indicates highly significant differences.)

The phenotypes of transgenic *A. thaliana* plants were significantly different not only in the aerial parts but also in the root system (Figure 4A–I). The root weight and lignin content were measured at the same location in *A. thaliana* roots. Compared with those of the WT plants, the root weights of the 35S:*At NAC20* and RC *At NAC20* plants decreased by 36.33% and 62.99%, respectively, and their lignin contents significantly increased by 57.29% and 66.36%, respectively. However, the root weight of *At nac20* increased by 41.95%, and the lignin content decreased significantly, by 27.71% (Figure 4J,K). Safranin O/fast green staining of the cross-sections of the infiltrated roots of transgenic *A. thaliana* revealed that 35S: *At NAC20* and RC *At NAC20* resulted in greater lignification and a higher root diameter, whereas the opposite trend in *At nac20* was observed. The above results further indicated that *AdNAC20* was involved in lignin biosynthesis and plant bolting and flowering growth and development.

### 2.4. AdNAC20 Increases Lignin and Reduces Coumarin in Transgenic ADF

#### 2.4.1. Generation of *AdNAC20*-Overexpressing Plants and Mutant Plants

**Figure 5:**
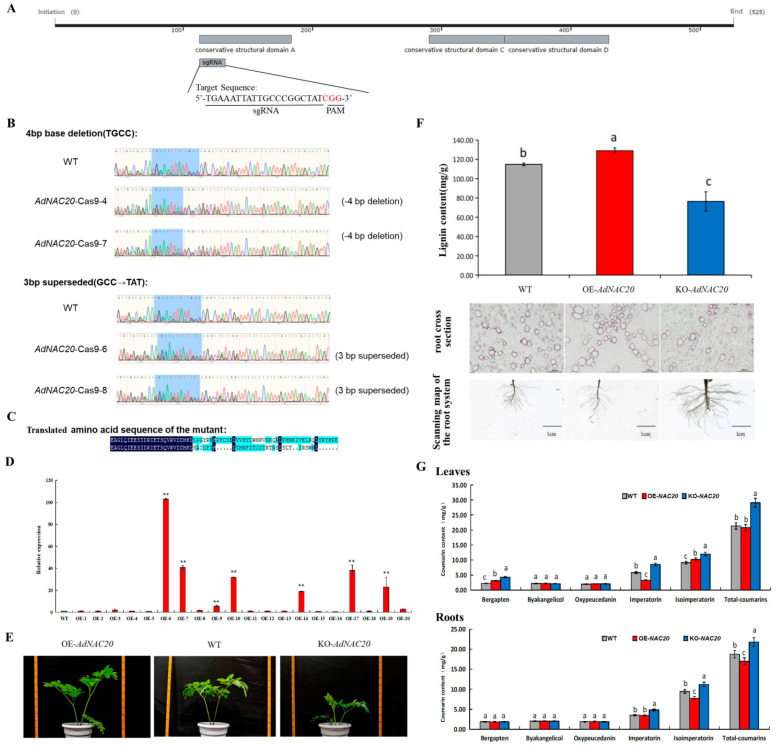
Identification of *AdNAC20 ADF* mutant plants via CRISPR/Cas9 mutagenesis. (**A**) Selection of sgRNAs for gene editing of *AdNAC20*. The gray boxes represent conserved structural domains; the straight lines extending out the region are the sgRNAs, and the red letters indicate the CGGs in the PAM sequence. (**B**) DNA identification of *AdNAC20* mutants in *ADF*. (**C**) Translated amino acid sequence of the mutant. (**D**) Expression of the *AdNAC20* gene in plants overexpressing this gene. (**E**) Phenotypes of the *AdNAC20*-modified lines. (**G**) Coumarin content in the roots and leaves of gene-edited *ADF* and the control. (**F**) Effective lignin extraction in the *AdNAC20*-modified lines. Section observation of root lignin phloroglucinol staining of *ADF* roots. Phloroglucinol redifies lignin, and the darker the color is, the greater the degree of lignification. OE-*NAC20*, *AdNAC20*-overexpressing plants of *ADF*; KO-*NAC20, AdNAC20* mutant plants of *ADF*; WT, wild-type plants of *ADF*. (** indicates highly significant differences.)

Six OE-*NAC20* and seven KO-*NAC20* plants were obtained via the transformation of *A. tumefaciens* strains harboring 35S:*AdNAC20* and *AdNAC20-Cpf1* construct vectors (Figure 5A–E). Six mutant plants (KO-*NAC20*) were obtained, among which four positive transgenic lines were identified by PCR analysis, and the editing efficiency was 66.67%. The reason for these results was that two of the mutant plants had a 4 bp base deletion, while the other plants had a 3 bp base substitution in the target region (Figure 5B). In other words, the base sequence of the edited mutant plants was translated into incorrect proteins that produced a stop codon in advance (Figure 5C). The gene expression of the *AdNAC20*-overexpressing *ADF* plants increased, and the transformation efficiency was 35%. The *AdNAC20* expression levels of the OE-6, OE-7, OE-9, OE-10, OE-14, OE-17, and OE-19 plants were significantly different (103.01, 40.72, 5.46, 31.63, 18.79, 38.13, and 22.99 times, respectively) (Figure 5D). The results showed that the *AdNAC20* gene was transcribed and expressed normally in the transgenic *ADF* plants.

#### 2.4.2. Comparative Phenotypes, Coumarin and Lignin Determination of Transgenic ADF

By comparative analysis, the plant height of the OE-*NAC20 ADF* plants was higher than that of the KO-*NAC20* and WT plants (Figure 5E). The degree of root lignification in the OE-*NAC20* treatment group was greater than that in the WT group, the lignin content significantly increased by 12.28%, and the root diameter significantly decreased. KO-*NAC20* had the opposite effect, and the lignin content of KO-*NAC20* decreased by 33.48% compared with that of the WT. The above results were consistent with those of heterologous expression in *A. thaliana*. The overexpression of *AdNAC20* increased the lignin content and the degree of lignification in the plants, and vice versa in the mutant plants. (Figure 5F).

Furthermore, compared with that in the roots of the WT plants, the coumarin content in the roots of the OE-*NAC20* plants, as determined by HPLC, was 17.01 mg/g, an increase of 9.28%. The concentration in the KO-*NAC20* roots was 21.8 mg/g, which was a 16.3% increase. There was no significant difference in the imperatorin content between OE-*NAC20* and WT roots, but the imperatorin content of KO-*NAC20* plants reached 4.84 mg/g, which was significantly different from that of WT plants. The isoimperatorin content of OE-*NAC20* roots (7.77 mg/g) was significantly lower than that of WT plants (9.43 mg/g), but the isoimperatorin content of KO-*NAC20* plants (11.20 mg/g) was significantly higher than that of WT plants. There was no significant difference in the contents of oxypeucedanin, bergapten, and byakangclicol in OE-*NAC20* roots compared with those in WT plants, but the content of bergapten in KO-*NAC20* leaves was significantly higher than that in OE-*NAC20* and WT plants (Figure 5G). The results showed that the *AdNAC20* gene not only promoted the lignin biosynthesis of *ADF* but also decreased the biosynthesis of coumarin, especially isoimperatorin, imperatorin, and bergapten.

### 2.5. Transcriptome Data Analysis of Transgenic ADF Plants

#### 2.5.1. Functional Annotation and Classification of ADF Unigenes

Through transcriptome sequencing of *ADF* seedlings, the obtained transcriptome unigene proteins were annotated to multiple databases, such as UniProt, NR, Pfam, Rfam, eggNOG, GO, and KEGG (Table S7). Then, the DEGs were analyzed using the EBSeq R package, whose thresholds were set as follows: FC > 2 and *p* < 0.05. A total of 29,671 DEGs were identified (Table 1, Figure S1A). After KO annotation, an intergroup comparison between the transgenic *ADF* and WT control groups revealed that a large number of DEGs were enriched in the phenylpropanoid pathway according to KEGG pathway analysis (Figure S1B,C), indicating that the *AdNAC20* gene was related to phenylpropanoid pathways.

#### 2.5.2. DEG Analysis

##### Analysis of DEGs Associated with the Biosynthesis of Coumarin and Lignin Metabolism in ADF Phloem

**Figure 6:**
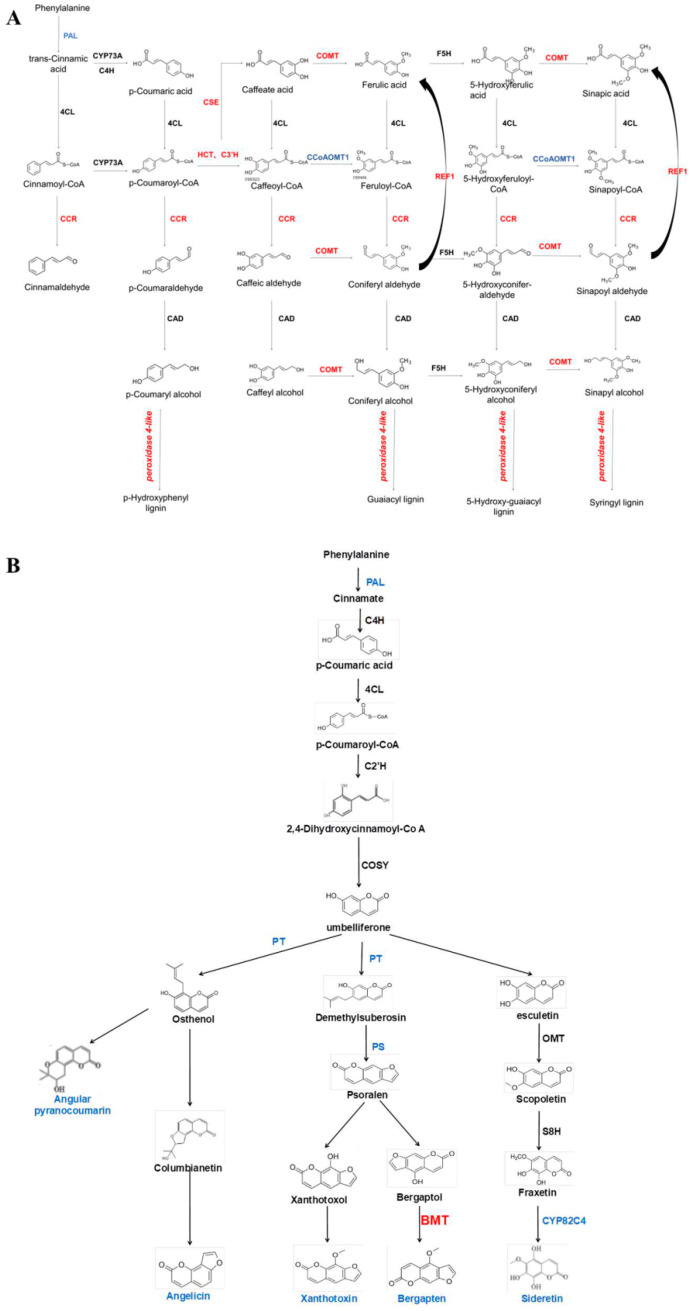
*AdNAC20* upregulated *lignin*-related enzyme-encoding genes and downregulated coumarin-related enzyme-encoding genes in the phloem. Red font indicates up, blue font indicates down, and black font indicates no significant change. (**A**) *AdNAC20* upregulated lignin-related enzyme-encoding genes in the phloem. (**B**) *AdNAC20* downregulates coumarin-related enzyme-encoding genes in the xylem. PAL, phenylalanine ammonia-lyase; CYP73A, trans-cinnamate 4-monooxygenase; C4H, cinnamate-4-hydroxylase; 4CL, 4-coumarate--CoA ligase; CSE, caffeoylshikimate esterase; C3H, coumarale-3-hydroxylase; COMT, caffeic acid 3-O-methyltransferase; CCoAOMT1, caffeoyl-CoA 5-O-methyltransfenase; CAD, cinnamyl-alcohol dehydrogenase; CCR, cinnamoyl-CoA reductase; REF1, coniferyl-aldehyde dehydrogenase; F5H, ferulate-5-hydroxylase; PT, prenyltransferase; PS, psoralen synthase; BMT, bergaptol O-methyltransferase; OMT, O-methyl-transferase; CYP82C4, 5-hydroxy-8-methoxypsoralen; C2H, coumarale-2-hydroxylase; COSY, coumarin synthase; S8H, scopoletin 8-hydroxylase; HCT, shikimate O-hydroxycinnamoyltransferase. (Red font indicates up-regulation of expression, blue font indicates down-regulation of expression, black font indicates no significant change in expression.)

**Figure 7:**
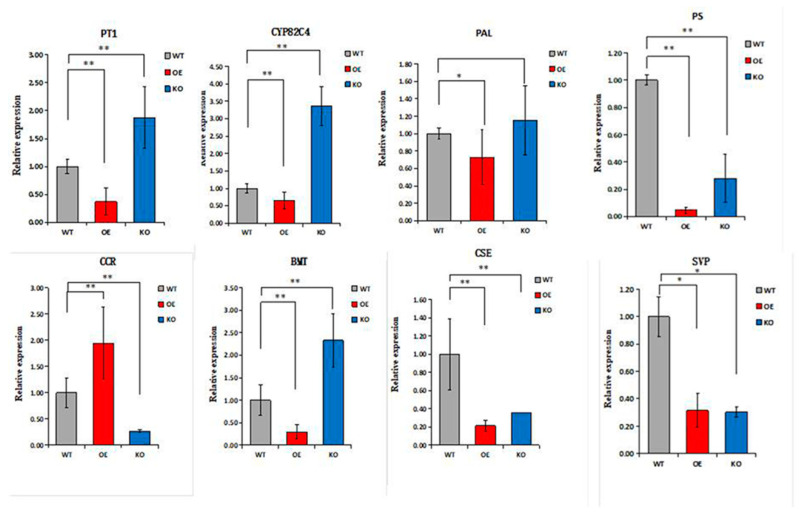
Expression levels of related genes in *ADF* xylem. * Indicates a significant difference, and ** indicates an extremely significant difference. OE, *AdNAC20*-overexpressing plants of *ADF*; KO, *AdNAC20* mutant plants of *ADF*; WT, wild-type plants of *ADF*; PAL, phenylalanine ammonia-lyase; PT, prenyltransferase; PS, psoralen synthase; CCR, cinnamoyl-CoA reductase; BMT, bergaptol O-methyltransferase; CSE, caffeoylshikimate esterase; CYP82C4, 5-hydroxy-8-methoxypsoralen; SVP, short vegetative phase.

The biologically active compounds were mainly distributed in the root phloem of *ADF*. In this study, DEGs in the root phloem associated with lignin and coumarin biosynthesis in the phenylalanine pathway were analyzed *(*Figure 6A,B). *AdNAC20* expression was negatively correlated with the expression of *PAL*, a cocatalytic enzyme of lignin and coumarin biosynthesis. CCR, COMT, peroxidase 4-like, HCT, C3H, and REF1, which are associated with lignin biosynthesis, were positively correlated with *AdNAC20* expression, while CCoAOMT was repressed. CCoAOMT participates not only in lignin biosynthesis but also in several biosynthetic pathways leading to soluble phenylpropanoids. The S/G ratio is a major determinant of lignin quality [10]. CCoAOMT deficiency has a small effect on lignin content and acts mainly in the biosynthetic pathway of G monomers, causing an increase in the S/G ratio [10]. In addition, CCoAOMT also plays a role in coumarin biosynthesis in *A. thaliana* roots [8]. *AdNAC20* negatively regulated the coumarin biosynthesis genes PT, PS and CYP82C4. The average logarithmic values of DEGs associated with coumarin and lignin biosynthesis in the leaves, root xylem, and root phloem of the *ADF* treatment were also consistent with the aforementioned results. To verify the DEGs associated with *AdNAC20*, eight genes were selected for qPCR analysis, and the measured results were consistent with the transcriptome results and demonstrated the reliability of the transcriptome data (Figure 7).

##### Analysis of DEGs with Opposite Expression Patterns in the Xylem and Phloem of ADF

**Figure 8:**
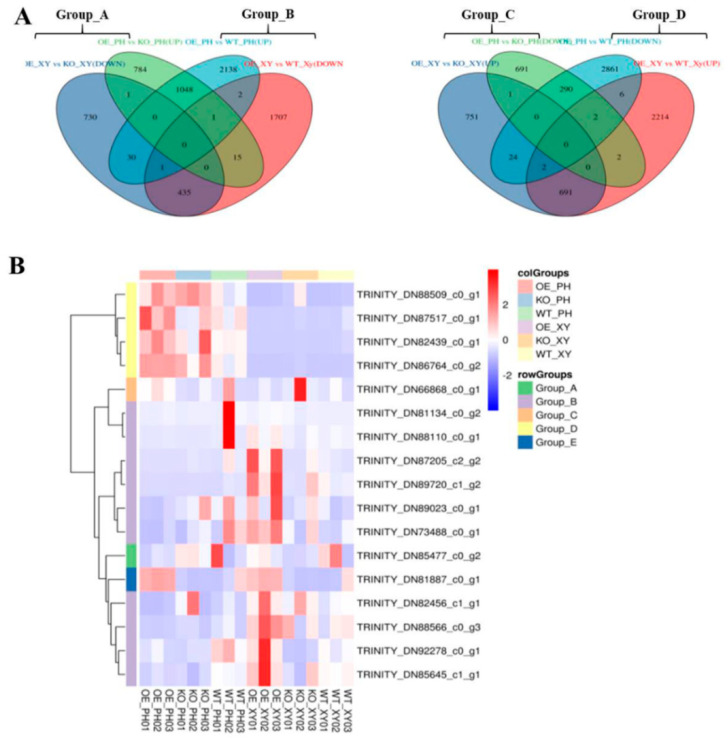
DEGs with opposite expression patterns in the xylem and phloem of *AdNAC20* transgenic plants analyzed via a Venn diagram. (**A**) DEGs of 16 genes divided into four groups, Groups A~D. (**B**) Heatmap of DEGs of the four groups and *AdNAC20* as Group E. OE_PH, root phloem of *AdNAC20*-overexpressing plants of *ADF*; KO_PH, root phloem of *AdNAC20* mutant plants of *ADF*; WY_PH, root phloem of wild−type plants of *ADF*; OE_XY, root xylem of *AdNAC20*-overexpressing plants of *ADF*; KO_XY, root xylem of *AdNAC20* mutant plants of *ADF*; WY_XY, root xylem of wild-type plants of *ADF*.

DEGs with opposite expression patterns in the xylem and phloem of *AdNAC20* transgenic plants were analyzed via a Venn diagram, and the 16 mined genes were divided into four groups: Groups A~D (Figure 8A,B). Homologous sequences of *TRINITY_DN85477_c0_g2* (*RHOGDI*) of Group A and *TRINITY_DN73488_c0_g1* (*PID*) and *TRINITY_DN88566_c0_g3* (*NRT1*) in Group B are reportedly involved in signal transduction and auxin transport in plant roots [25,26,27,28,29]. The gene *TRINITY_DN89023_c0_g1* in Group B was annotated as a phosphotransferase (EIIC) and methyl-CpG binding domain (MBD). This gene is associated with the phase transition from vegetative to reproductive in the meristem, and its homologous sequence has been reported to be involved in sugar transport [30], assuming that it provides energy for plant development from vegetative to reproductive. *TRINITY_DN82456_c1_g1 (BRG3*) in Group B is a C3HC4-type (RING finger) protein with a ubiquitination function. Some studies have shown that *BRG3* delays tomato ripening [31] and represses gibberellin responses in *A. thaliana* [32] *TRINITY_DN88110_c0_g1* (*Pbs27*) in Group B was related to photosystem II chlorophyll. Its homologous sequence is involved in the process of physiological photosynthesis [33]. The NAC transcription factor *TRINITY_DN89720_c1_g2* (*NAC7*-like) of Group B is a homologous sequence of DCAR_027802 in *Daucus carota* L. var. *sativa*. Notably, *TRINITY_DN86764_c0_g2* (*REF1*) and *TRINITY_DN88509_c0_g1* (*BMT*) in Group D were annotated in the phenylpropane pathway. Both genes, 2.31- and 2.66-fold, respectively, were upregulated in the root phloem of OE-*NAC20* but downregulated in the root xylem of OE-*NAC20* compared with WT. Previous studies have shown that *REF1* is involved in ferulic acid and sinapic acid biosynthesis to promote lignin biosynthesis in *A. thaliana* [34]. *BMT* is involved in the biosynthesis of bergapten, which belongs to coumarins [35,36]. It has been reported that PS downregulation inhibits the generation of psoralen, and *BMT* downregulation inhibits the production of bergapten by psoralen [35].

##### Protein-Protein Interaction Analysis of *AdNAC20*

**Figure 9:**
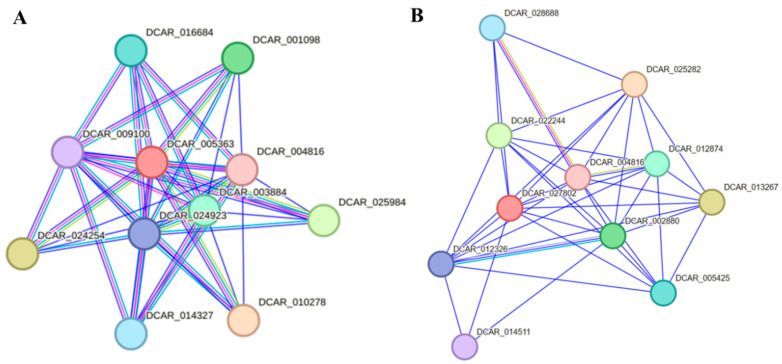
Protein–protein interactions of proteins predicted using STRING 12.0 software based on the *Daucus carota* L. var. *sativa* protein database. (**A**) Interaction network of DCAR 005363, a homologous protein of *AdNAC20*. (**B**) Interaction network of DCAR_027802, a homologous protein of *TRINITY_DN89720_c1_g2* (*NAC7-like*).

*AdNAC20* was identified as a homologous protein of DCAR_005363 (XP_017233235.1, *NAC68-like*) from Section 2.2.1 above. Protein–protein interactions of DCAR 005363 and its related genes were predicted using STRING 12.0 software based on the *Daucus carota* L. var. *sativa* protein database (Figure 9A) [37]. The 10 predicted functional partners of DCAR 005363 were annotated with functional information for only four genes. DCAR_003884 and DCAR_004816 are auxin response factors (ARFs) that bind specifically to auxin-responsive promoter elements (AuxREs). DCAR_016684 is a methyltransferase. DCAR_009100 is an LRRNT_2 domain-containing protein. These genes might interact with *AdNAC20*. In addition, DCAR_004816 (*ARF*) was predicted to be a functional partner of both DCAR 005363 and DCAR_027802 (*NAC7-like*) and has been reported to be an auxin response factor using STRING 12.0 software (Figure 9B).

## 3. Discussion

*ADF* is an important herbal raw material of traditional Chinese medicine and a commonly used flavor and spice in China [4]. The main herbal raw material of *ADF* is dried roots, in which the phloem scatters secretory tissue oil tubes, which are the main site of coumarin accumulation [3,38]. This study also showed that coumarins are mainly found in the phloem of *ADF* roots (Figure 1). Bolting is an important physiological process of the phase transition from vegetative to reproductive growth [5]. However, the early bolting of *ADF* will affect its yield and quality [5,39]. In this study, when the number of *ADF* leaves reached nine or ten, the phloem/xylem ratio in roots decreased, the thickness of secondary cell walls increased, the lignin content of the root phloem increased significantly, and the coumarin content of the root phloem decreased significantly during the early bolting of *ADF (*Figure 1). In contrast to changes in root xylem, the changes in the root phloem were more significant during the early bolting of the ADF.

According to previous transcriptome data [24], *AdNAC20* was cloned from ADF. The NAC domain of *AdNAC20* was not conserved, and subcellular localization experiments verified that *AdNAC20* was located in the nucleus and other parts of the cell, indicating the functional richness of the *AdNAC20* gene (Figure 2). With further transient transformation of the ADF roots, the lignin content of OE-*NAC20* increased significantly, and the coumarin content decreased significantly, while KO-*NAC20* had the opposite effect (Figure 3). The above results suggested that *AdNAC20* functions via dual pathways that positively promote lignin formation and negatively regulate coumarin formation.

The homologous expression of *AdNAC20* was performed in *A. thaliana* to quickly verify the function of the gene (Figure 4) [40]. The overexpression of *AdNAC020* reduced the root weight, root diameter, lignin content and bolting and flowering time of *A. thaliana* but increased the plant height of *At nac20* plants, which was similar to the early-bolting phase of ADF. Then, the ADF plants were transformed via the floral-dip method. The phenotypes and lignin content of OE-*NAC20* of ADF were consistent with those of 35S:*NAC20* of *A. thaliana*, and the *AdNAC020* gene regulated coumarin biosynthesis of ADF, especially bergapten and isoimperatorin (Figure 5). These results further indicated that the *AdNAC20* gene regulated lignin and coumarin biosynthesis in the phenylpropanoid pathway in ADF roots.

Phenylpropanoids contribute to stimulus responses in plants [8,9]. PAL, C4H and 4CL are the cocatalytic enzymes of lignin and coumarin biosynthesis in the phenylpropanoid pathway in most plants. *AdNAC20* expression was higher in the root phloem than in the leaf, stem, and root xylem in the ADF treatment (Figure 2D). Based on the transcriptome data and qRT-PCR analysis of the ADF root phloem, coumarin biosynthesis genes, such as CYP82C4, PS, and PT, were inhibited, but lignin biosynthesis genes, such as COMT, CCR, and peroxidase 4-like, were increased. The results were also verified by CRISPR, whose patterns were complementary (Figure 6). In the ADF roots, lignin was mainly distributed in the xylem, and coumarin was mainly distributed in the phloem (Figure 1). Sixteen genes were found by the analysis of the opposite DEGs in the xylem and phloem (Figure 8), among which TRINITY_DN86764_c0_g2 (REF1) and TRINITY_DN88509_c0_g1 (BMT) were annotated in the phenylpropanoid pathway. BMT is involved in catalyzing all the hydroxylated coumarin in *P. praeruptorum* and is a paralogous homologous sequence of COMT involved in lignin biosynthesis [41]. According to the transcriptome data of the present study, BMT had 11 transcripts. Based on the above analysis, we speculated that TRINITY_DN88509_c0_g1 (BMT) regulated the biosynthesis of coumarin and lignin, and different transcripts had different functions, which needs to be validated prospectively.

**Figure 10:**
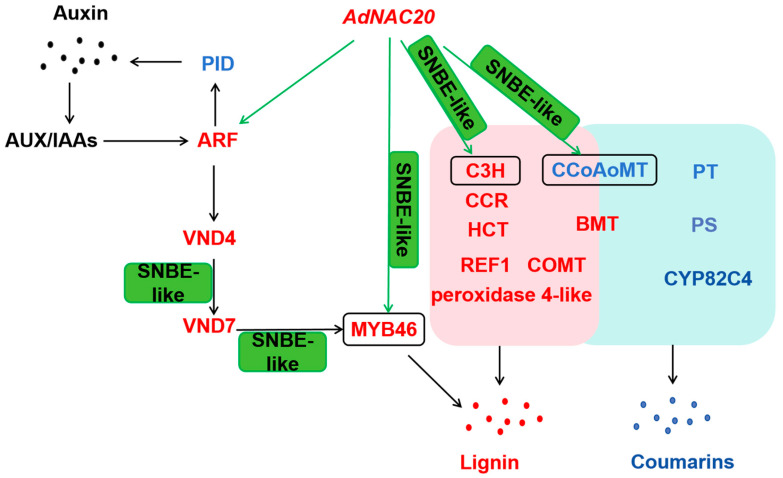
Schematic diagram of the gene network interacting with *AdNAC20* during early-bolting of *ADF*. *AdNAC20* may be involved in the transcriptional network of lignin biosynthesis controlled by auxin and may also regulate lignin and coumarin biosynthesis via the phenylpropanoid pathway. SNBE-like indicates that a gene regulates the expression of the next gene by recognizing this motif in the promoter. Green arrows represent the predicted direct transcriptional regulation of *AdNAC20* in this study. Red font represents upregulated genes. The blue font represents downregulated genes. The pink background represents the lignin biosynthesis pathway. The blue background represents the coumarin biosynthetic pathway. AUX, auxin response factors; IAAs, indole-3-acetic acid induced proteins; VND4, vascular-related NAC domain protein 6; VND7, vascular-related NAC domain protein 7; PID, protein kinase PINOID 2-like; ARF, auxin response factor; PAL, phenylalanine ammonia-lyase; CYP73A, trans-cinnamate 4-monooxygenase; C4H, cinnamate-4-hydroxylase; 4CL, 4-coumarate--CoA ligase; CSE, caffeoylshikimate esterase; C3H, coumarale-3-hydroxylase; COMT, caffeic acid 3-O-methyltransferase; CCoAOMT1, caffeoyl-CoA 5-O-methyltransfenase; CAD, cinnamyl-alcohol dehydrogenase; CCR, cinnamoyl-CoA reductase; REF1, coniferyl-aldehyde dehydrogenase; F5H, ferulate-5-hydroxylase; PT, prenyltransferase; PS, psoralen synthase; BMT, bergaptol O-methyltransferase; OMT, O-methyl-transferase; CYP82C4, 5-hydroxy-8-methoxypsoralen; C2H, coumarale-2-hydroxylase; COSY, coumarin synthase; S8H, scopoletin 8-hydroxylase; HCT, shikimate O-hydroxycinnamoyltransferase. (Red font indicates up-regulation of expression, blue font indicates down-regulation of expression.)

By sequence alignment and phylogenetic tree construction with NAC transcription factors in *A. thaliana* and ADF [24], DCAR_027802 (NAC7-like), a homologous sequence of TRINITY_DN89720_c1_g2, was found to be most closely related to *A. thaliana* AT1G12260.1 (VND4) (Figure S2). VND4 is specifically expressed in the secondary xylem in the root–hypocotyl region [15], which was consistent with the upregulation of TRINITY_DN89720_c1_g2 in *AdNAC20*-overexpressing ADF xylem. Studies have shown that VND1–7 regulates secondary wall biosynthesis by activating the expression of SNBE-like motifs [15]. The SNBE, TERE, and X1E1 motifs are palindromic sequences comprising four CTT/AAG sequences with 2 bp intervals (CTTNNCTTNNAAGNNAAG), which is the ‘Ideal Core Structure for binding by VND7′ (ICSV) [42]. SNBEs are present in the promoters of CgMYB46, CgC3H, and CgCCoAOMT in Citrus grandis [37]. MYB46 and MYB83 act as the master switches regulating secondary wall biosynthesis, and the NAC-MYB-GRN model of developmental lignin biosynthesis was established in *A. thaliana* [43,44]. In this study, AdMYB46 expression in the ADF xylem of OE-*NAC20* plants was significantly higher than that in the WT and KO-*NAC20* plants, and AdMYB46 expression in the xylem was higher than that in the phloem of OE-*NAC20* plants. In this study, we speculated that a possible regulatory pathway of *AdNAC20* is associated with the auxin regulation of lignin and coumarin biosynthesis (Figure 10), which will provide a reference for future experiments. *AdNAC20* was predicted to directly regulate the transcription of several genes with SNBE-like motifs in their promoter, such as MYB46, C3H, and CCoAOMT. NAC proteins bind to DNA as dimers [15,45,46]. The CCoAOMT and BMT genes were predicted to regulate both lignin and coumarin biosynthesis in ADF. *AdNAC20* may also interact with NAC family proteins and regulate other differentially expressed genes. In conclusion, *AdNAC20* regulated both lignin and furocoumarin biosynthesis in ADF roots, and further studies on the specific mechanisms by which *AdNAC20* regulates lignin and coumarin biosynthesis are urgently needed for early bolting of ADF.

## 4. Materials and Methods

### 4.1. Plant Materials and Growth Conditions

In this study, we initially determined that *AdNAC20* has an important function by conducting experiments in *A. thaliana*, and then returned to *ADF* for more in-depth studies. Seeds of *ADF* variety ‘Chuanzhi No. 2’ were sown under substrate formula conditions without fertilization (peat:vermiculite = 2:1). The seedlings were cultured in the greenhouse of Sichuan Agricultural University, China. The seeds of *A. thaliana* (Col-0) and *Nicotiana benthamiana* were directly sown in pots under substrate formula conditions without fertilization (peat:vermiculite:perlite = 1:1:1). *A. thaliana* seeds were kept at 4 °C for 3 d to break dormancy. *A. thaliana* and *ADF* were grown on soil in a growth chamber (16 h light/8 h dark) at 25 °C after stratification. For *N. benthamiana* only, the pots were grown on soil in a growth chamber (16 h light/8 h dark) at 25 °C. All experiments were performed in triplicate and repeated at least three times.

### 4.2. Histological Analysis

The samples were fixed for 48 h, dehydrated in an ethanol series (25, 50, 75, 95 and 100% (*v*/*v*)) for 15 min at each dilution, dipped in 1/2 xylene + 1/2 anhydrous ethanol, made transparent to pure xylene, made 1/2 paraffin + 1/2 xylene and pure paraffin wax, and embedded in wax. The embedded materials were sliced on a microtome (Leica RM2016). The paraffin sections were deparaffinized with water, subjected to environmentally friendly transparent solution I for 20 min, environmentally friendly transparent solution II for 20 min, absolute ethanol I for 5 min, absolute ethanol II for 5 min and 75% (*v*/*v*) ethanol for 5 min, and finally washed with running water. The slices were stained with phloroglucinol A, B, and C for 5 s, 30 s, and 1 min, respectively, to visualize the roots. After absorbing part of the dye with a straw, the slices were sealed with a cover glass, and photos were taken within 3 min. A WinRHIZO root analysis system was used to scan the *ADF* root system, and the scanning data were analyzed with supporting analysis software.

### 4.3. Coumarin and Lignin Determination

Lignin content was determined using a plant lignin ELISA kit (Camaisu Biotechnology Company, Shanghai, China), and double-antibody one-step sandwich ELISA was used to determine lignin content. This procedure was carried out according to the manufacturer’s instructions.

The coumarin content was determined by high-performance liquid chromatography (SHIMADZU LC-16, SHIMADZU Corporation, Kyoto, Japan). Separation was carried out on an C18 column (250 mm × 4.6 mm, 5 μm; Agilent Technologies Inc., Santa Clara, CA, USA) set at 30 °C using a mobile phase consisting of ultrapure water (A) and acetonitrile (B). The gradient program was as follows: 0–8 min, 5–20% (B); 8–40 min, 20–55% (B); 40–55 min, 55–95% (B); and 55–58 min, 95–5% (B) using a 1 mL/min flow rate. Detection was performed at 300 nm. Each sample was analyzed for 60 min. Twenty microliters of standard solution was injected into an HPLC system and separated. Three biological replicates were analyzed for each genotypic plant, with each biological replicate repeated three times.

The plant materials were washed, surface-dried, and then dried at 50 °C in a 101-3A electric blast drying oven (Beijing Zhongxing Weiye Instrument Co., Ltd., Beijing, China) to a constant weight. After being chopped, the samples were pulverized by an FW135 grinder (Shanghai Wujiu Automation Equipment Co., Ltd., Shanghai, China) and placed in a desiccator. The samples were passed through a 100-mesh sieve, approximately 0.1 g was accurately weighed with an ISO9001 electronic balance (SARTORIUS, Goettingen, Germany), 20 mL of methanol was added to a 50 mL centrifuge tube, and the tube was shaken well. The mixtures were extracted for 60 min using an SB25-12DTDN ultrasonic cleaner (Ningbo Xinzhi Biotechnology Co., Ltd., Ningbo, China, 300 W, 40 kHz). Then, methanol was used to quantitatively. The suspended particles were preliminarily filtered through double-loop qualitative filter paper (Ge Biotechnology Co., Ltd., Shanghai, China) and eventually filtered through a 0.45 μm filter (Shanghai Xinya Purification Device Factory, Shanghai, China) to obtain sample solutions. The peak area external standard method (ESTD) was used for quantitative determination. The standard solutions were prepared as follows. To obtain stock solutions, 2.70 mg of oxypeucedanin, 3.42 mg of isoimperatorin, 3.43 mg of imperatorin, 1.13 mg of bergapten, and 1.61 mg of byakangclicol were added to methanol in five 5 mL volumetric bottles. Then, the above stock solutions were transferred to another five 5 mL volumetric bottles, diluted with methanol to volume, mixed and stored at 4 °C. The mixed solutions were diluted 1, 2, 4, 8, 16, 20, 40, 100, and 200 times with methanol and set aside.

### 4.4. RNA Extraction, Gene Cloning and qRT-PCR

Total RNA was extracted using TRIzol reagent (TIANGEN, Beijing, China). Using a HiScript^®^II Q RT SuperMix reverse transcription kit (Vazyme, Nanjing, China), total RNA was reverse-transcribed according to the manufacturer’s instructions. Primer 5 5.0 software was used to design the primer pairs for *AdNAC20*. 2×Taq PCR Mix (TIANGEN, China) was used to clone the *AdNAC20* gene. The pClone007-*AdNAC20* fusion construct was generated using a pClone007 Blunt Vector Kit (Tsingke, Beijing, China). The fusion construct was detected by DNA sequencing. Each qRT-PCR was performed using SYBR^®^ Premix Ex Taq™ II (TaKaRa, Beijing, China). The primers used for qRT-PCR are listed in Table S1.

### 4.5. Plasmid Construction

All of the constructs used were verified by DNA sequencing. *AdNAC20* in *ADF* was cloned and transformed into the pCAMBIA3301 vector (modified pCAMBIA3301-35S-eGFP-kana) under the control of the CaMV35S promoter (35S::*AdNAC20*) using a ClonExpress II One Step Cloning Kit (Vazyme, China). The primers used are shown in Table S2. Cas9/GRNA-phosphine oxammonium vector (Catalog. No. VK005-15 (Beijing Weishang Lide Co., Ltd.) was used to construct the *AdNAC20* CRISPR/Cas9 vector (*AdNAC20-Cpf1*). To generate CRISPR/Cas9 *mutant* lines, *20 bp* of the AdNAC20 exon was *selected*, and specific sgRNA sequences were designed by the website (http://skl.scau.edu.cn/targetdesign/, (accessed on 15 June 2024)), and the sgRNA sequence was 5′-TGAAATTATTGCCCGGCTAT-3′. The gRNA sequences of primers used were 5′-TTGTGAAATTATTGCCCGGCTAT-3′ and 5′-AACATAGCCGGGCAATAATTTCA-3′. The Cpf1/gRNA-GFP vector (Catalog. No. VK005-206, Beijing Weishang Lide Co., Ltd., Beijing, China) was used.

### 4.6. Subcellular Localization Analysis

CELLO v. 2.5 (http://cello.life.nctu.edu.tw/ (accessed on 1 May 2019)) was used for the subcellular localization prediction of genes. *Agrobacterium* suspensions (*Agrobacterium tumefaciens* strain GV3101) were added to YEB + Kana + Rif liquid media at 28 °C and cultured at 180 rpm to an OD_600_ of 0.8–1.0. The suspensions were centrifuged and then resuspended in MMA (5% sucrose, 10 mM MgCl_2_, 10 mM MES, 100 μM AS, 0.05% Silwet L-77) (OD_600_ = 0.8–1.0). Leaves from 3-week-old *N. benthamiana* and *ADF* plants with true leaves were infiltrated through their abaxial surfaces with *Agrobacterium* suspension in a greenhouse. At 2–3 days post infiltration in the dark, the whole leaf tissue at the infiltration sites was collected and examined using a Leica confocal microscope (A1-90i) (Leica Microsystems (Shanghai) Trading Co., Shanghai, China).

### 4.7. Transient Expression of ADF Roots

The transient expression method was described in the research of Yang et al. [47]. The verified pCAMBIA3301-*NAC20*-GV3101 and *AdNAC20-Cpf1*-GV3101 *Agrobacterium tumefaciens* constructs were added to YEB + Kana + Rif liquid media at 28 °C and cultured at 180 rpm to an OD_600_ of 0.8–1.0. Agrobacterium solutions were centrifuged, resuspended in MMA (100 mM MES, 100 mM MgCl_2_, AS, Silwetl-77, pH 5.7, ddH_2_O) at an OD_600_ of 0.8, and finally seeded into 1.5 mL sterile enzyme-free EP tubes. Seedlings of the same growth size were selected, and their root system was washed, dried, and then soaked in packaged bacterial solution in the dark for 5 days. The infiltrated roots were imaged using a Leica confocal microscope.

### 4.8. Plant Transformation

The 35S::*AdNAC20* and *AdNAC20-Cpf1* constructs were individually transformed into the *E. coli* strain DH5α via a freeze–thaw method. After sequence confirmation, the fusion constructs 35S::*AdNAC20* and *AdNAC20-Cpf1* were introduced into *A. tumefaciens* strain GV3101. The *A. thaliana* gene AT5G50820.1 was highly homologous to *AdNAC20*. Then, mutant seeds of AT5G50820.1, an *AdNAC20* mutant line of *A. thaliana* (*At nac20*), were purchased from AraShare for PCR validation. Primers were designed according to the T-DNA Primer Design website (http://signal.salk.edu/tdnaprimers.2.html (accessed on 13 May 2021)). *Agrobacterium* suspensions were used for steady transformation via the floral-dip method to obtain the overexpression lines (35S:*NAC20*) and reverse-complemented lines (RC *At NAC20*) of *A. thaliana* plants [48]. T3 homozygous lines of *A. thaliana* were used for phenotypic investigation. The verified pCAMBIA3301-*NAC20*-GV3101 and *AdNAC20-Cpf1*-GV3101 *Agrobacterium* suspensions were *steadily* transformed into *ADF* plants via the same floral-dip method [48].

### 4.9. Illumina Sequencing and Differentially Expressed Gene (DEG) Analysis

Illumina sequencing was used to detect *AdNAC20*-overexpressing plants (OE-*NAC20*), *AdNAC20* mutant plants (KO-*NAC20*), and wild-type “Chuanzhi 2” plants (WT) of *ADF* under normal conditions, which were divided into leaf, root phloem, and root xylem, respectively. All the samples were divided into 9 groups (Table S3). Each group contained three biological replicates. High-quality RNA libraries were sequenced on a DNBSEQ-T7-PE150 sequencing platform at MGI Tech Co., Ltd. (Shenzhen, China). Transcriptome assembly and gene functional annotation were performed at SeqHealth Tech Co., Ltd. (Wuhan, China). Raw sequencing data was first filtered by Trimmomatic 0.36, low-quality reads were discarded and the reads contaminated with adaptor sequences were trimmed. The remaining reads of each sample were subjected to transcriptome assembly using Trinity. Seven public databases, including UniProt, Rfam, Pfam, NR, KEGG, GO, and eggNOG, were used for functional annotation of each unigene. Gene expression was calculated using the reads per kilobase per million mapped reads (RPM) method to compare differences between different samples. Differentially *expressed* genes (DEGs) were screened using the following criteria: |log_2_ (FC) > 1| and a *p* value < 0.05. GO enrichment analysis (http://www.geneontology.org/ (accessed on 17 April 2023)) and KEGG enrichment analysis (http://www.genomejp/kegg/ (accessed on 17 April 2023)) were performed using KOBAS 2.1.1 software. We utilized transcriptome tools (e.g., Venn, Heatmap, DESeq2, etc.) from Wuhan SeqHealth Tech Co., Ltd., Wuhan, China, for deeper transcriptome data mining. The protein-protein interactions of genes were predicted using STRING 12.0 software [37].

### 4.10. HPLC Analysis

Coumarins content was determined by high-performance liquid chromatography (SHIMADZU LC-16, SHIMADZU Corporation, Kyoto, Japan). HPLC-grade methanol was purchased from Xilong Scientific Co., Ltd., Shantou, China. Ultrapure water was obtained from Wahaha pure water. The gradient elution was conducted in C18 column (250 mm × 4.6 mm, 5 μm; Agilent Technologies Inc., USA) with the mobile phase of ultrapure water (A) and acetonitrile (B) at a flow rate of 1.0 mL·min ^−1^ and the column temperature of 30 °C. The applied gradient program was as follows: 0–8 min, 5–20% (B); 8–40 min, 20–55% (B); 40–55 min, 55–95% (B); 55–58 min, 95–5% (B) using 1 mL/min flow rate. Detection was analyzed at 300 nm. Each sample was analyzed for 60 min. An amount of 20 μL of standard solutions were injected into a HPLC system and separated. Three biological replicates were analyzed for each genotypic plant, with each biological replicate repeated three times.

## 5. Conclusions

Early-bolting *ADF* had significantly greater degrees of root lignification and lower coumarin contents than that of the normal plants. In this work, we cloned a NAC transcription factor gene (*AdNAC20*) from *Angelica dahurica* var. *formosana* (*ADF*). It was shown to play a dual pathway function that positively enhanced lignin formation but negatively controlled coumarin formation. It was predicted to directly regulate the transcription of several genes with SNBE-like motifs in their promoter, such as MYB46, C3H, and CCoAOMT. And the heterologous expression of the *AdNAC20* gene at *A. thaliana* proved that the *AdNAC20* gene plays an important role in the process of bolting and flowering.

## Figures and Tables

**Table 1:** Number of differentially expressed genes in the transcriptome.

Tissue	OE vs. WT		KO vs. WT		OE vs. KO	
	UP	DOWN	UP	DOWN	UP	DOWN
Leaf	1664	1420	1613	944	913	1002
Root phloem	3220	3185	1377	1644	1849	966
Root xylem	2917	2161	998	1132	1469	1197

## Data Availability

Data were contained within the article and Supplementary Materials.

## Supplementary Materials

The following supporting information can be downloaded at: https://www.mdpi.com/article/10.3390/ijms25147998/s1.
