# Supplementary material for: AdNAC20 Regulates Lignin and Coumarin Biosynthesis in the Roots of Angelica dahurica var. Formosana
Source: Int J Mol Sci. 2024 Jul 22;25(14):7998. doi: 10.3390/ijms25147998 (PMC11276817; doi:10.3390/ijms25147998)
Supplement: Supplementary file 1 [file ijms-25-07998-s001.zip › ijms-3085018-supplementary.pdf]

International Journal of *Molecular Sciences*

MDPI

Article

AdNAC20 regulates lignin and coumarin biosynthesis in the roots of *Angelica dahurica* var. *formosana*

Wenjie Qu [1†], Wenjuan Huang[1, 2†], Chen Chen[1], Jinsong Chen[1], Lin Zhao[1], Yijie Jiang[1], Xuan Du[1], Renlang Liu[1], Yinyin Chen[1], Kai Hou[1], Dongbei Xu[1], Wei Wu[1*]

[1] College of Agronomy, Sichuan Agricultural University, Chengdu 611130, Sichuan, P. R. China; qwj1941428057@163.com (W.Q.); 15754368373@139.com (W.H.); DoubleChen_SWUN@yeah.net (C.C.); chjs1993@163.com (J.C.); linzhao0518@outlook.com (L.Z.); jyj177ky@163.com(Y.J.); 15729872162@163.com (X.D.); liurenlang2023@163.com (R.L.) 14227@sicau.edu.cn (Y.C.) hking@sicau.edu.cn (K.H.); xudongbei2006@126.com(D.X.)

[2] Sichuan Academy of Agricultural Characteristic Plants, Sichuan Academy of Agricultural Sciences, Neijiang 641200, Sichuan, P. R. China; 15754368373@139.com(W.H.)

* Correspondence: ewuwei@sicau.edu.cn (W.W.); Tel.: +86-138-8028-6059

†These authors contributed equally to this work and share first authorship

1. Supplementary Figures and Tables

1.1. Supplementary Tables

Supplementary Table S1 Primers for genes cloning

PCR

Sequence name	Unigene	Forward primer (5'−3')	Reverse primer (5'−3')
AdNAC020	TRINITY_DN81887_c0_g1	TGATTATGGAGGAAAGTGA	TCCGAATGTTGTTTATGG
AdPAL	TRINITY_DN92718_c3_g1	CTGAGGACCAAGCCACTGAG	AGCCGAAGTCATGTCAGCAA
AdCYP82C4	TRINITY_DN88210_c0_g1	ACCATGCACACCAGTACCAG	ACTGGCGCGAGATACGTAAG
AdPT1	TRINITY_DN81739_c0_g1	CGGCCATAGAAAATGCAGCC	GCGAAGGGAATGCGATTGTC
AdBMT	TRINITY_DN88509_c0_g1	AACTTGCACACTGGTGTCCA	CGATGCACCCATCATGCTTG
AdCSE-like	TRINITY_DN89673_c4_g1	AATTTCCGGCAAGATCCCGT	GCTTTGATTCGCCCGTCATC
AdCCR	TRINITY_DN91420_c0_g7	CAGGATTTCCACCACCTCCC	TCACCGGTGCAGTTAAGACC

Supplementary Table S2 Homologous primers for *AdNAC20* overexpressed vector and CRISPR/Cas9 vector

Sequence name	Unigene	Forward primer (5'−3')	Reverse primer (5'−3')
AdNAC020	TRINITY_DN81887_c0_g1	gaacacgggggactctacgtaATGGAGGA AAGTGATATCAAGGTGC	ccgcctgaaccgcctccaccGTCTTGTG TCCTCTTGACAATTACAGG

Supplementary Table S3 Samples name of transcript of *A. dahurica* var. *formosana*

Tissue	Plant type		
	OE-*NAC20*	WT	KO-*NAC20*
Leaf	OE_LE	WT_LE	KO_LE
Root phloem	OE_XY	WT_XY	KO_XY

Root xylem	OE_PH	WT_PH	KO_PH

Supplementary Table S4 *AdNAC20* cDNA sequence and protein

Sequence name	Unigene	cDNA sequence	protein
AdNAC020	TRINITY_DN81887_c0_g1	ATGGAGGAAAGTGATATCAAGGTGCAACATGACAATAGTGAATATGAGGCAGGCCTGCAAATTGAAGAAAGTATCGACAGAATTGAAACTTCTCAAGTGCGTGTTGACGACATGAAATTATTGCCCGGCTATCGGTTTCATCCATTTGATTATGAACTAGTAGTTCATTACTTGTGGAACAAGGTGAACAAACAGCCTCTCCCTCACAATAAGATCGTGGAACTTAAACAGCTTTACAAGTATCATCCAGAGGAAATTACAAAAACAGACCAGGGATTGGTAGAGAATGAGTGGTACTTTTTCACAGAGACGGAGAATGTGCAACTGGTGATGGTTACTGGAAAGCCACTGAAGATGAAGAAACGGTGTATTATAAAGGTGTTGCCGTTGGACATAGGAAGGAATTTGTGTGTTATCGAGGAAAAGCTTTTCCGCCAAAAGGAGACGAGACGAACTGGATCTTGCATGAATTTACAGTCACTGCATGTCCAAGTACCTGTAATTGTCAAGAGGACACAAGACTAG	MEESDIKVQHDNSEYEAGLQIEESIDRIETSQVRVDDMKLLPGYRFHPFDYELVVHYLWNKVNKQPLPHNKIVELKQLYKYHPEEITKTDQGLVENEWYFFTETENVQLVMVTGKPLKMKKRCIIKVLPLDIGRNLCVIEEKLFRQKETRRTGSCMNLQSLHVQVPVIVKRTQD

Supplementary Table S5 Physicochemical properties of NAC genes of *A. dahurica* var. *formosana*

Gene Name	Gene ID	ORF	Number of amino acids	PI	Unstable index	Fatty coefficient	GRAVY
AdNAC020	CL25188Contig1	525	174	6.32	62.59	91.67	-0.586

Supplementary Table S6 Secondary helical structure of NAC genes of *A. dahurica* var. *formosana*

Gene Name	Alpha helix (Hh)	3_{10} helix (Gg)	Pi helix (Ii)	Beta bridge (Bb)	Extended strand (Ee)	Beta turn (Tt)	Bend region (Ss)	Random coil(Cc)
AdNAC020	31.61%	0.00%	0.00%	0.00%	22.99%	10.34%	0.00%	35.06%

Supplementary Table S7 Number of Unigene for different database annotations

DataBase	AnnotatedNumber
Uniprot	186238
NR	190310
Pfam	190908
Rfam	278505

DataBase	AnnotatedNumber
eggNog	164136
GO(GO_term; class; description)	138761
KEGG(ko; class; description)	51441
Total	298837

1.2. Supplementary Figures

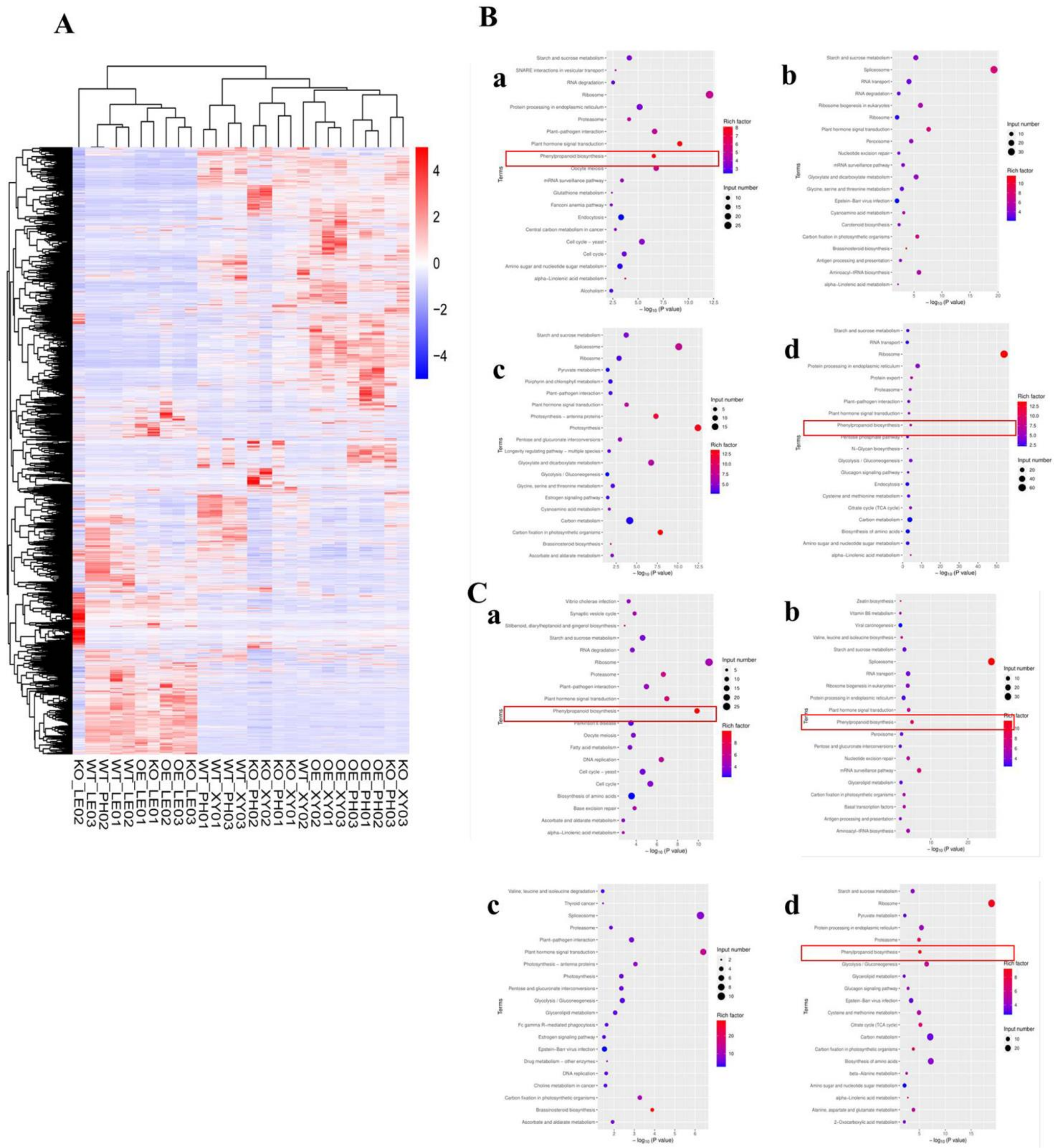

Supplementary Figure S1 KEGG annotation and classification of the transcriptome Unigene. (A) Global hierarchical clustering heat map of differentially expressed genes. (B) KEGG analysis in phloem of *A. dahurica* var. *formosana.* (a) up-regulation of OE vs WT;(b) Down regulation of OE vs WT; (c) KO vs WT down-regulated; (d) KO vs WT was upregulated. (C) KEGG analysis of DEGs in the xylem of *A. dahurica* var. *formosana.* (a) up-regulation of OE vs WT; (b) Down regulation of OE vs WT ; (c) KO vs WT down-regulated; (d) KO vs WT was upregulated. OE, *AdNAC20*-overexpressing plants of *ADF*; KO, *AdNAC20* mutant plants of *ADF*; WT, wild-type plants of *ADF*.

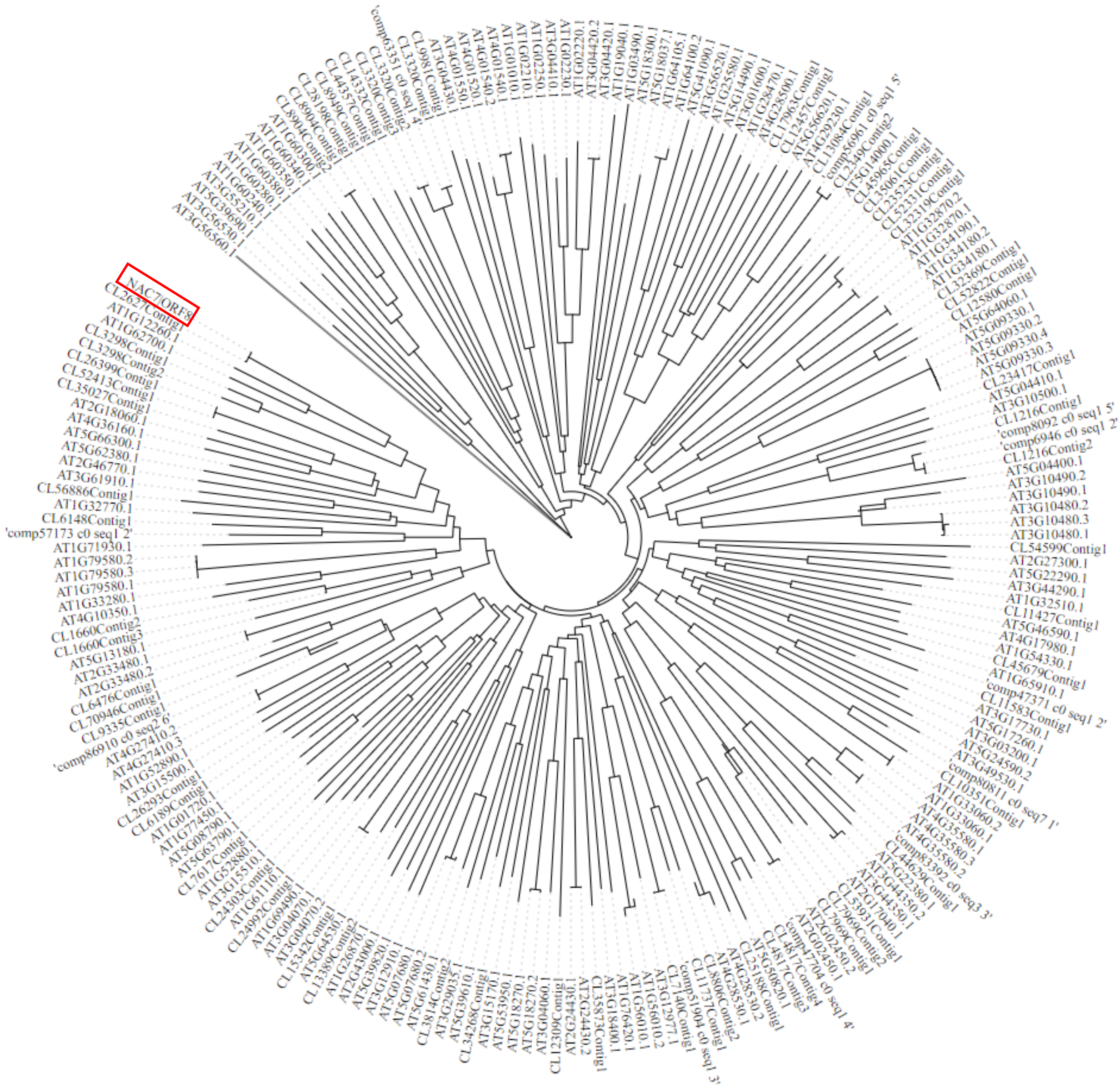

Supplementary Figure S2 By sequence alignment and phylogenetic tree construction with DCAR_027802 (*NAC7*-like) and NAC transcription factors in *Arabidopsis* and *A. dahurica* var. *for-mosana.*
